# Fractal analysis highlights analogies in arenaceous tubes of *Sabellaria alveolata* (Metazoa, Polychaeta) and agglutinated tests of foraminifera (Protista)

**DOI:** 10.1371/journal.pone.0273096

**Published:** 2022-08-26

**Authors:** N. Mancin, F. dell’Acqua, M. P. Riccardi, G. Lo Bue, A. Marchini

**Affiliations:** 1 Dipartimento di Scienze della Terra e dell’Ambiente, Università di Pavia, Pavia, Italy; 2 Dipartimento di Ingegneria Industriale e dell’Informazione, Università di Pavia, Pavia, Italy; Universita degli Studi di Urbino Carlo Bo, ITALY

## Abstract

Bioconstructions of *Sabellaria alveolata* (Polychaeta Sabellariidae) from southern Sicily (Central Mediterranean) were sampled and analysed through a multidisciplinary approach in order to unravel the construction pattern of arenaceous tubes and explore possible analogies existing between the worm tubes and the agglutinated tests of benthic foraminifera (Protista). Scanning Electron Microscopy and Energy Dispersive Spectroscopy analyses were carried out on entire tubes as well as sectioned ones. Results show that arenaceous tubes are built following a rigorous architectural framework, based on selection and methodical arrangement of the agglutinated grains, and show surprising analogies with the test microstructure previously observed in agglutinated foraminifera. The grain distribution detected in both model species bioconstructions was analysed using a fractal numerical model (Hausdorff fractal dimension). Collected data show that in both organisms the grains were distributed according to a fractal model, indicating that the evolutionary process may have led to finding the same optimal constructive strategy across organisms with an independent evolutionary history, notwithstanding different geometrical scales. Furthermore, in sectioned tubes we observed microplastic fragments agglutinated within the arenaceous wall and in the inter-tube area. This unexpected finding shows that marine animals can be affected by microplastic pollution not only in soft tissues, but also engineered hard structures, and suggests the problem is more pervasive than estimated so far.

## Introduction

Agglutination is a mechanism used by some aquatic protozoans and metazoans to bind particles to build an external case that serves for protection of soft body parts, or for crypsis. This strategy appeared early on in evolutionary history, at least since the early Phanerozoic over 500 millions of years ago [e.g. 1–5]. Grains are either selected or randomly picked from the water column or bottom sediment, then manipulated in three dimensions and cemented to form the agglutinated wall. This ability has been observed in unicellular organisms, such as tintinnids, testate amoebae and agglutinated foraminifera [e.g., 6–20], as well as in different multicellular groups, such as arthropods (tubicolous amphipods and tanaids, as well as caddisflies) and polychaete annelids [5, 21–29].

Several agglutinated foraminifera, primarily the genera belonging to the Order Textulariida [e.g. *Textularia* Defrance, *Karreriella* (Cushman), *Colominella* Popescu, *Eggerella* Cushman] and Lituolida [e.g. *Vulvulina* d’Orbigny, *Navarella* Ciry & Rat and *Spiroplectammina* Cushman], precisely select sediment grains to build arenaceous tests using pseudopodia. In these taxa, the constructive pattern is mainly characterized by selected grains arranged according to a layered microstructure. The different layers of the wall are often formed by mineral particles of different compositions [12, 18, 30–32 and references therein]. These agglutinating foraminiferal genera, although coming from different geological contexts, show the same test building strategy, indicating that grain selection is based on genetics and not exclusively controlled by environmental adaptations. This observation is reinforced by the fact that grain selection can persist during foraminiferal test growth or can change after early growth stages, as a function of compositional variations in bottom sediments, or as a genetically controlled shift in behaviour from juveniles to adults [e.g. 12–18, 30, 32–34].

Grain selection and their spatial distribution within the foraminiferal agglutinated wall has been reported to follow a fractal numerical model, probably adopted by foraminifera to optimize the production of cement and to make the agglutinated test more resistant [7, 35]. A similar grain selection capability can be expected in metazoans such as polychaete worms, which are also equipped with specialized organs for sorting and manipulating grains and for secreting bio-adhesive organic cement [22, 36–39]. Whether grain arrangement in polychaete-made tubes follows a fractal model like in agglutinated foraminifera had not yet been investigated.

Here, we apply Scanning Electron Microscopy (SEM) and Energy Dispersive Spectroscopy (EDS) analyses, followed by fractal analysis to investigate the tube-building strategy of the honeycomb worm *Sabellaria alveolata* (Linnaeus, 1767). This sabellariid polychaete is a common sedentary, intertidal reef-builder of Atlantic and Mediterranean coasts [40]. In Italy, it mostly occurs along the Tyrrhenian coast and in southern Sicily, where it builds wave-resistant reefs made on sand-sized grains [41]. Not all grains are the same: they differ in several chemical-mineralogical properties, as well as in buoyancy performance; *S*. *alveolata* selects them when they are suspended by wave motion according to their local abundance, size and shape, showing a certain preference for bioclastic remains [41–46].

SEM-EDS observations performed on sectioned aggregated tubes are here used to compare the patterns of sand grains arrangement in the polychaete tubes with those occurring in the microscopic tests of a selected species of agglutinated foraminifera. To this purpose, we chose as model species *Karreriella novangliae* (Cushman), a deep-water textulariid foraminifer coming from Pleistocene records of the Pacific Ocean [18]. Sectioned specimens of *K*. *novangliae* and of other agglutinated foraminiferal species had been previously studied by Mancin et al. [18] to unravel test microstructure and to assess whether the grain selection process was controlled by environmental conditions or by genetics. This work uses the same methodology and instruments utilised in this previous study, thus we are ensured the acquired microstructural data are comparable. Fractal analysis, on the other hand, is conducted on both *S*. *alveolata* and *K*. *novangliae* for the first time in this research.

## Materials and methods

### Sample collection, preparation and microscope analysis

*Sabellaria alveolata* bioconstructions were sampled from two sites of Southern Sicily (Italy, Central Mediterranean sea): Santa Barbara (36°46’49”N; 14°31’57”E) and Porto Turistico (36°46’53”N; 14°32’35”). At both locations, veneer type bioconstructions *sensu* [38] consisting of small patches growing on rocky shores in the intertidal zone, at a water depth varying from 0 to 1 m. Sampling permissions were not required, since the bioconstructions occurred in areas not subject to restrictions, and the target invertebrates (polychaete worms) are not included in the Directive 2010/63/EU of the European Parliament on the protection of animals used for scientific purposes. Additionally, we collected only a few, minute portions of bioconstructions (about 15–20 aggregated tubes), paying attention to avoid damaging the reef. *Sabellaria alveolata* bioconstructions are naturally subject to wave erosion [41, 44], which is much more destructive than our removal of small portions.

At the time of sampling during summer 2017, reefs were in good condition, without evident degraded or eroded parts, and with living polychaetes in the tubes. In each site, two portions of bioconstruction were collected using a steel spatula and immediately stored in sterile glass jars containing 70% ethanol. In the laboratory, after the verification of taxonomic identity of ethanol-preserved polychaete specimens observed under a dissecting microscope, samples were cleaned of the worm remains using metal tweezers, dried in an oven at about 40°C for two days and prepared according to the methodology described by Mancin et al. [31]. Each portion was mounted on a stub covered by a carbon conductive adhesive tape. Then, samples were carbon-coated for morphological analyses (*in*Beam technique) by Scanning Electron Microscope (SEM, Tescan FESEM, series Mira 3XMU), at the CISRiC Arvedi Laboratory (University of Pavia). After this first step, the same bioconstruction portions were embedded in epoxy resin and cut along both horizontal and vertical planes (S1 Fig, available online as supporting information) in order to evaluate possible changes in tube diameter, wall thickness and size, shape and composition of the agglutinated grains during the tube building process. Finally, the sections (a total of 9) were polished with diamond pastes (diamonds varying from 0.25 to 6 μm in grain size), carbon-coating and analysed using the SEM equipped with x-ray Energy Dispersive Spectroscopy (EDS). Back Scattered Electron (BSE) images of sectioned tubes highlighted compositional similarities (or dissimilarities) among agglutinated grains through the arenaceous wall thickness, on the basis of the mean atomic number of each grain forming the agglutinated tube. The elemental compositions of the single grains forming the agglutinated wall were provided through standardless spot microanalyses. In order to check the chemical-mineralogical variability of the agglutinated grains within the sectioned wall of each tube, two-dimensional x-ray maps of selected elements were also collected. In each elemental map, the colour intensity is proportional to the element concentration in each image pixel (in black areas the element is lacking), thus the comparison of the maps from the same area of the sample provided an overview of element distribution in that area [31 and references therein]. Multiple maps for silicon, calcium and aluminium were simultaneously acquired in order to discriminate the different mineralogical phases that make up the arenaceous grains. In particular, the elemental maps visually indicate the content of calcium (the major constituent of carbonates as calcite and dolomite), silicon (the major constituent of quartz) and aluminium (that, together with Si, characterizes feldspars) in the agglutinated grains.

Microplastic particles, unexpectedly encountered during analyses of sectioned tubes, were identified through BSE imaging and electron microanalysis [e.g. 47–50]. For elemental quantification, reference spectra (e.g. epoxy resin and quartz) with sufficient x-ray counts were collected as well as spectra of suspected microplastics. The spectra were subsequently processed by applying the appropriate ZAF (Z-atomic number, A-absorption and F-fluorescence correction) procedure. The peculiar morphology of the microplastic particles, often displaying surfaces with cracks and pits, and their chemical composition allowed us to distinguish microplastics from other organic particles, since plastics are carbon-based but with smaller amounts of other elements, such as chlorine, sulphur, silicon, titanium, etc. [50].

### Fractal analysis

Fractal dimension is commonly accepted as a statistical index of complexity, reflecting how detail in a pattern changes with the scale at which it is measured [51]. As such, it can be used to compare complex structures of significantly different scales. In order to evaluate whether the agglutinated grains forming the arenaceous tubes of *S*. *alveolata* and the agglutinated tests of *K*. *novangliae* follow a similar distribution in space (e.g. the agglutinated wall), the local Hausdorff fractal dimension was calculated on a square sliding window on back-scattered electron images acquired at the SEM, thus generating a map of fractal dimension over the entire image. The pixel intensity was taken as the third spatial dimension necessary to compute the Hausdorff dimension, and the size of the window was set at 32 pixels as a compromise between resolution and statistical significance.

Firstly, the whole map of grain distribution was calculated in the area corresponding to the wall forming an arenaceous tube, as well as a portion of agglutinated wall forming a test chamber. Secondly, horizontal and vertical transepts, that crossed both agglutinated walls, were considered by plotting the position in the transept (*x* axis) against the Hausdorff fractal dimension (*y* axis).

## Results

### Polished sections and chemical elemental maps

**Fig 1:**
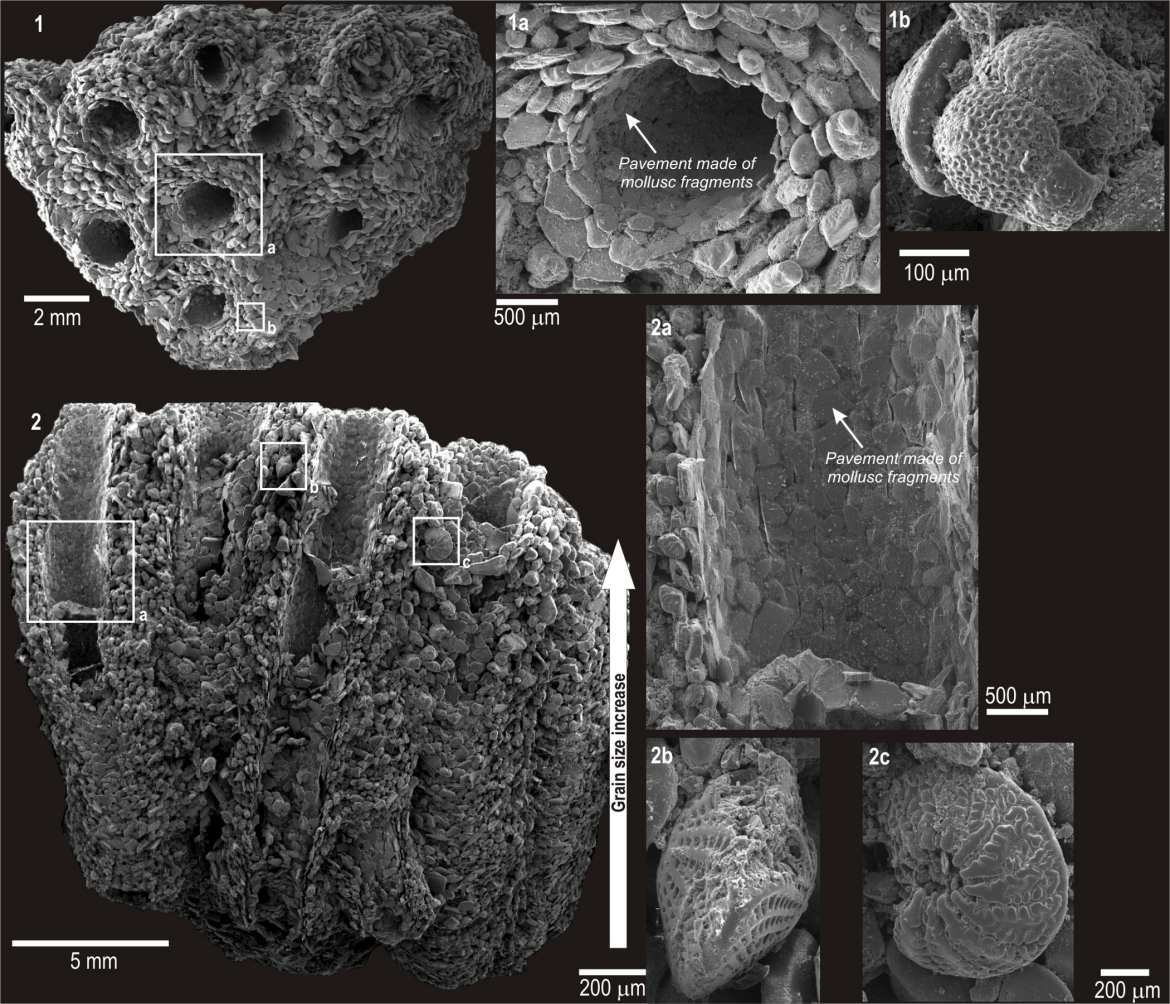
SEM images in secondary electrons of *Sabellaria alveolata* bioconstruction from site SB. 1. Horizontal view showing the typical honeycomb structure; 2. Vertical view highlighting the internal structure of tubes covered by a smoothed pavement.

*Sabellaria alveolata* bioconstructions from both sites were characterized by regularly agglutinated tubes of different diameters forming the honeycomb structure typical of this species (Fig 1, photo 1 and S4 Fig, photo 1).

**Fig 2:**
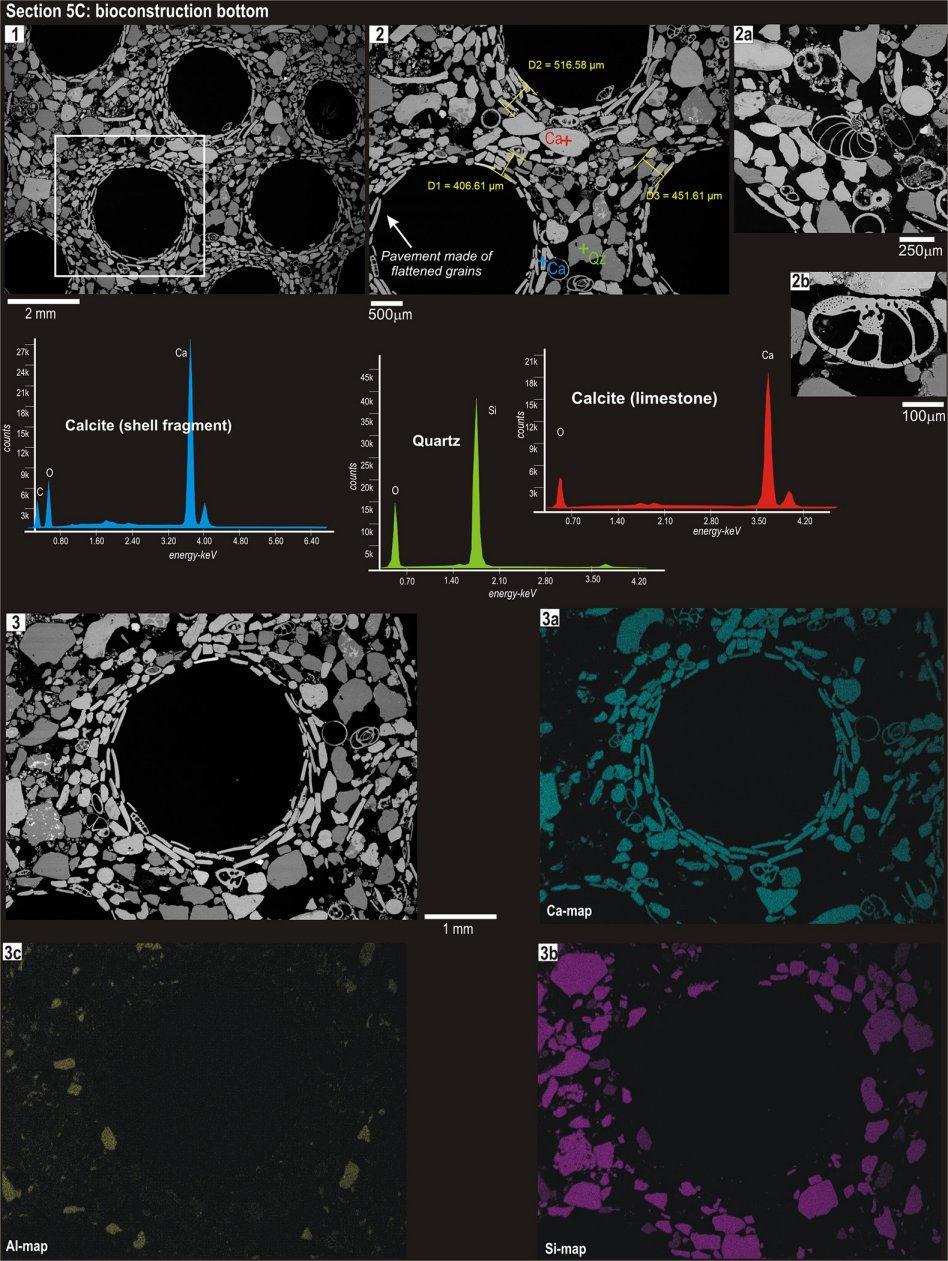
Back-scattered electron (BSE) image of aggregated tubes (the same of Fig 1; photo 1) horizontally sectioned at the bottom. The coloured crosses indicate the spots for standardless microanalyses; the corresponding EDS spectra as well as the elemental maps (silicon-Si in violet, aluminium-Al in yellow and calcium-Ca in light blue) are reported below.

**Fig 3:**
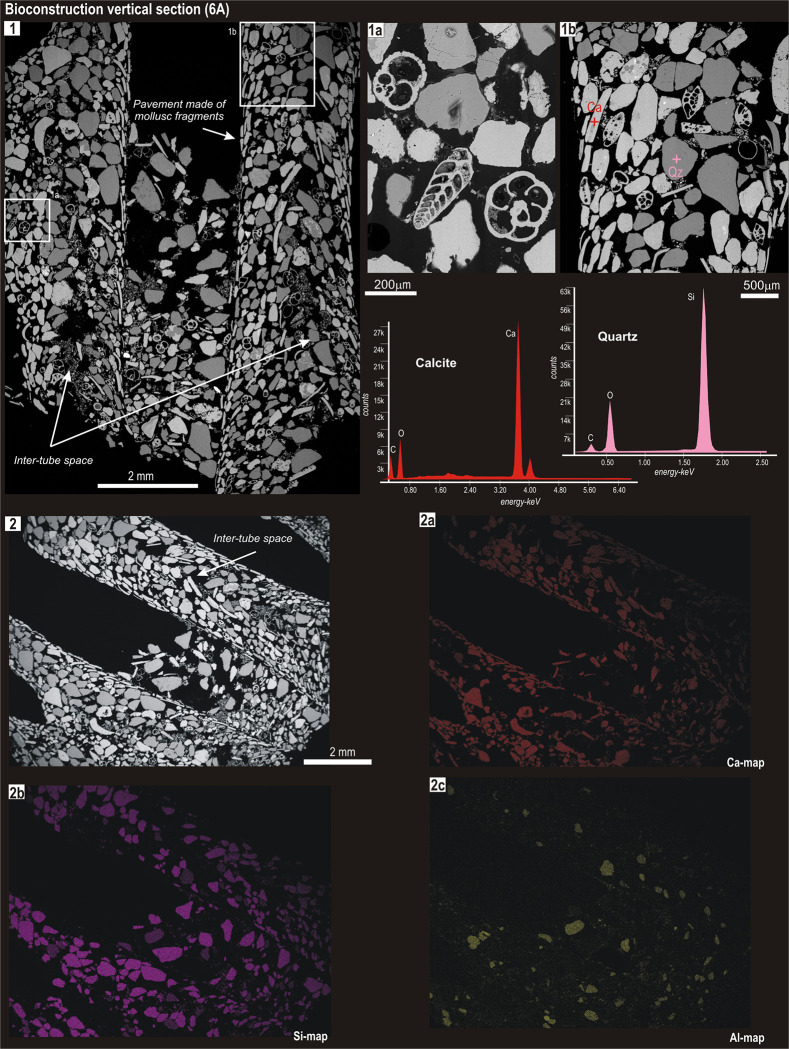
BSE image of aggregated tubes (the same of Fig 2; photo 2) vertically sectioned (6A). For further details in this figure, see also caption of Fig 2.

Each tube consisted of sand grains carefully selected and methodically arranged within the agglutinated wall according to a typical layered fabric: the smallest and flattest grains formed an inner lining that probably facilitates movement of the worm within the tube (Fig 1, 1A and 1B). The other grains, which also included several foraminiferal tests (1a, 2b, 2c), progressively increased in size moving outwards (1a). The grains also increased in size from the bottom of each tube upwards, following the polychaete growth direction (indicated by the large arrow in Fig 1). These features were more evident in sectioned tubes (Figs 2, 3 and S2–S5 Figs).

In horizontal sections (Fig 2 and S2–S4 Figs), the tube wall showed two distinct layers: inner and outer. The inner layer was thinner and made of flattened and equidimensional biogenic particles, tangentially orientated with respect to the internal cavity and forming a flat, smooth lining to the tube. The outer layer was thicker and formed by larger and more rounded grains made of lithic fragments and foraminiferal tests (Fig 2, 2A; S2 Fig, 1a). By contrast, inter-tube spaces did not show the typical layered disposition of the grains: these were randomly distributed and different in size, shape and composition (Fig 2, 1, 2; S2 Fig, 1 and S4 Fig, 2). Abundant foraminiferal tests, mainly with spherical and biconvex morphologies, occurred in both the outer part of the tube wall and the inter-tube space (Fig 2, 2A and 2B; S2 Fig, 1a). The tube wall thickness progressively increased up to the top of bioconstructions from both sites: it varied from about 400 to 600 μm at the bottom (Fig 2, 2; S4 Fig, 3), to ~600–700 μm in the middle portion (S2 Fig, 3) to over 1 mm at the top (S2 Fig, 1 and S4 Fig, 2, 3).

Vertical sections (Fig 3; S3–S5 Figs) showed an imbricated grain disposition, characterised by longer axes of grains that diverge outwards (Figs 3, 1 and 2; S3 Fig, 1, 2 and S5 Figs, 1, 2). This architectural frame persisted along the tube.

Standardless spot microanalyses carried out on single grains in both horizontal and vertical sections (e.g. Fig 2, photo 2; Figs 3 and 1) showed that the grains of the inner layer were made of calcite, probably remains of mollusc shells and echinoid spines or, more rarely, limestone fragments supplied by the rocky outcrops in the area. Conversely, the largest grains of the outer portion of the wall or filling the inter-tube space were mainly quartz and feldspar, along with a moderate amount of calcitic foraminiferal tests (e.g. Figs 3 and 2; S2 Fig, 1a).

The selection of agglutinated grains based on their composition becomes more evident when comparing the elemental maps of calcium-Ca, silicon-Si and aluminium-Al (e.g. Fig 2, images 3a-3c; Figs 3 and 2A–2C). Calcium, derived from limestone and calcareous biogenic material, was concentrated in the inner surface of the tube (the internal pavement of flattened grains), while Si and Al, derived from quartz and silicates, concentrated within the outer portion of the tube wall. In particular, Al grains were abundantly distributed in the inter-tube spaces (Figs 3 and 2C; S3 Fig, 2c). The chemical–mineralogical composition of the agglutinated grains was consistent within the different sections of a single tube, as well as across aggregated tubes, and across sites (Figs 2 and 3; S2–S5 Figs).

### Microplastics in *Sabellaria alveolata* tubes

**Fig 4:**
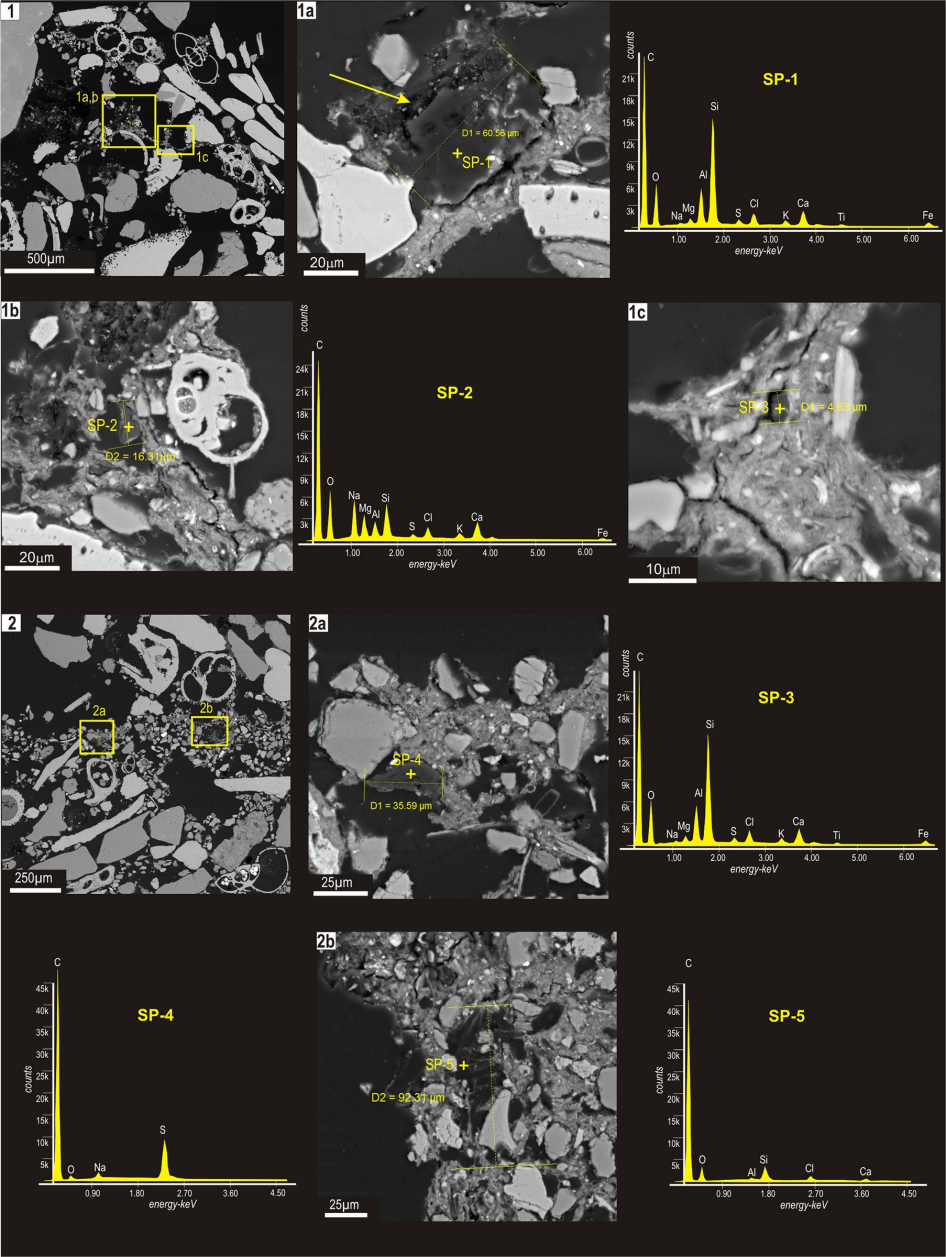
BSE images of microplastic particles and compositional spectra found in sectioned tubes from both sites.

Sectioned tubes of *S*. *alveolata* from both sites exhibited sporadic, small-sized particles of higher atomic weight (perceivable from their dark-grey to black colour) among grains composing the agglutinated wall and the inter-tubes spaces (Fig 4; S2 Fig, 1b and S3 Fig, 1a).

The particles were of variable shape and size, from a minimum of 4.5 μm to a maximum of about 92 μm, and with surfaces characterized by cracks and pits (Fig 4, arrow in 1a), likely due to oxidative weathering. The compositional spectra had a typical carbon peak, with minor amounts of Si, Al, S, Na, K, Ti, Fe and Cl, suggesting a probable plastic origin (Table 1).

**Table 1:** Compositional data relative to standardless spot-microanalyses performed on microplastic grains shown in Fig 4. Chemical data were recalculated to 100% and expressed in weight percent.

Spot-analysis	C	O	Na	Mg	Al	Si	S	Cl	K	Ca	Ti	Fe	Tot. (Wt%)
**SP—1**	64.8	14.8	0.3	0.5	2.2	9.9	0.2	0.5	0.6	4.5	0.2	1.5	100
**SP—2**	56.8	20.1	5.9	3.1	1.9	4.1	0.6	1.6	1.1	3.8	0.0	1.0	100
**SP—3**	58.8	16.6	0.2	0.6	3.7	11.4	0.6	1.7	1.0	2.9	0.3	2.2	100
**SP—4**	86.7	3.5	1.3	0.2	0.2	0.2	7.8	0.1	0.0	0.0	0.0	0.0	100
**SP—5**	72.2	17.0	0.1	0.2	0.9	6.2	0.1	1.3	0.3	1.0	0.1	0.6	100

The presence of Cl, the distinctive element of polyvinyl chlorine (PVC) [39], confirmed that some of those particles were in fact microplastics (Fig 4; SP-2, SP-3; SP-5; Table 1). Other particles had a peak in sulphur (Fig 4, SP-4; Table 1), that is characteristic of rubber. The very low concentration of elements, such as Si, Al, Na, K, Ti, Fe could be also due to the very fine-grained terrigenous component trapped within cracks and pits of microplastic fragments.

### Comparison with *Karreriella novangliae*

**Fig 5:**
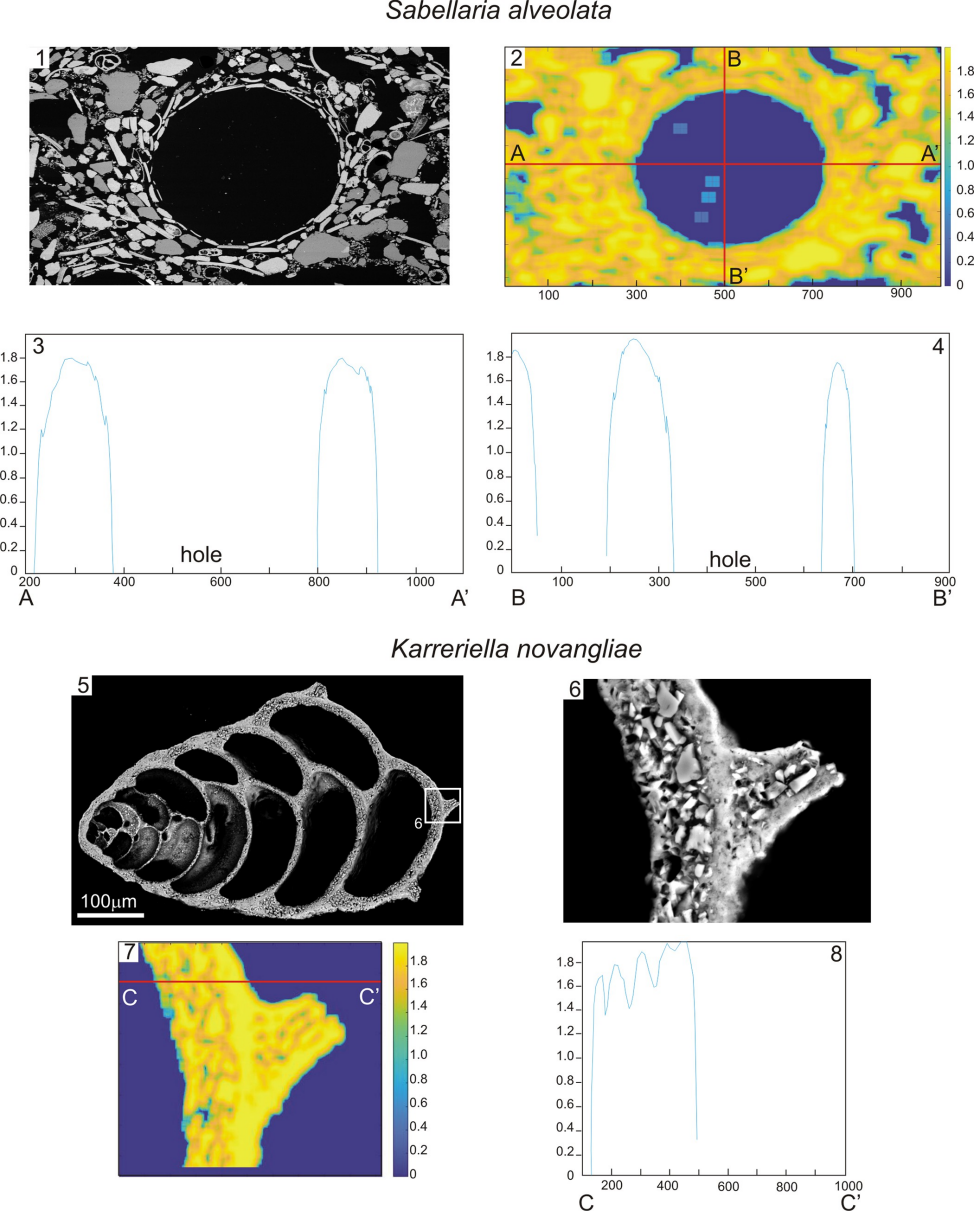
Mathematical distribution of the agglutinated grains in *Sabellaria alveolata* and *Karreriella novangliae* according to the fractal dimension of Hausdorff.

The grain distribution observed in *S*. *alveolata* areaneous tubes, as well as in the agglutinated foraminiferal tests matched a similar fractal model, although the Hausdorff dimension trends were slightly different (Fig 5).

In *S*. *alveolata*, moving outwards, a smooth change of the fractal dimension was observed within the wall thickness (Fig 5, images 3–4), while in *K*. *novangliae* the fractal dimension fluctuated more visibly (image 8), although presenting a similar, inverse-parabolae trend of values moving away from the chamber inside. By contrast, the ranges of values from both arenaceous walls overlapped quite well, both being between 1.4 and 1.8. More specifically, for the considered samples of *S*. *alveolata* and *K*. *novangliae* (obtained, respectively, on a series of 260 and 340 samples taken from SEM images), average values of Hausdorff dimensions across a representative transect were found to be 1.7501 and 1.6761, with standard deviations of 0.1693 and 0.2064 respectively.

## Discussion

### A “compositionally proven” selection of agglutinated grains

Results described above show that *Sabellaria alveolata* methodically selects sand-size grains, mostly with flattened and rounded shapes, to build its arenaceous tubes (Figs 1 and 2; S2–S5 Figs). The tube wall is formed by a layered microstructure with the smallest, flattest grains placed inside and the largest ones towards the tube margin; in the inter-tube area, grains are heterometric and chaotically arranged. The unprecedented acquisition of compositional maps of chemical elements demonstrates there are more calcium-based grains on the tube wall inside and more silicates towards the outside and inter-tube. This had been already observed and hypothesised for *Sabellaria* spp. [e.g. 43, 52, 53], but until this study had not been demonstrated.

Grain selection by *S*. *alveolata* takes place during the capture of sandy particles suspended in the water column by waves. Therefore, grains better adapted to buoyancy due to their shape, size and composition are reasonably more abundant in its tubes. For example, Lo Bue et al. [54] documented that the majority of the calcitic foraminiferal tests agglutinated in *S*. *alveolata* bioconstructions had spherical and biconvex morphologies, probably because they are more likely to persist in the water column for a long time, and hence be frequently available to the polychaete worm for capture. Heavy minerals have been observed to be very rare in sabellariid bioconstructions, even if abundant in the surrounding sea-floor sediment [55], because these grains do not float, but rather persist on the seabed, making them unavailable to the polychaete.

Calcitic grains, above all the ones with a biogenic origin, such as fragments of mollusc shells, are probably lighter than silicatic granules with the same size and shape, therefore can float more easily. Noteworthy is that in studied sectioned tubes, no micas (phyllosilicates with a typical leafy shape) were found, although we directly observed them in the surrounding sediment. Micas are thin and fragile, hence unsuitable to build wave-resistant, arenaceous tubes, although they are largely available for the worms.

In agglutinating organisms the grains are often selected on the basis of chemical-physical properties of the constituent mineral [12, 16, 56]. In foraminifera, for example, it has been shown that grains with high concentration of Zircon and Titanium are selected and agglutinated in specimens from high-energy environments, even when these heavy grains are very rare in the sediment [e.g. *Reophx nana* Rhumbler, from the Northern Adriatic Sea, 20; *Textularia hauerii* d’Orbigny, from the Barzaruto Archipelagos, 16]. Particularly, *R*. *nana* seems to use such heavy grains to stabilize the test within the sediment, when the sea-floor is swept by bottom currents [20]. Likewise, the infaunal foraminiferal species *Leptohalisi scottii* (Chaster) constructs an agglutinated test surface that mimics the flakes of fishes, exclusively using mica plates. This constructive pattern has been interpreted as an adaptative strategy to move quickly within the sediment, during short-term phytoplankton inputs at the sea-floor [20]. Our data seem to support a similar compositionally-based grain selection also in *S*. *alveolata*.

### Fractal models of sand grain arrangements

Other significant similarities seem to emerge from the comparison between the constructive strategies adopted by the polychaete worm *S*. *alveolata* and the model agglutinated foraminiferal species *K*. *novangliae*. The methodical arrangement of grains into a complex layered microstructure that follows a mathematical fractal model, already observed in unicellular foraminifera [11, 15, 35], and here confirmed for *K*. *novangliae*, is also firstly demonstrated here for sabellariid tubes as well. In particular, both organisms exhibit a surge of the Hausdorff’s fractal dimension in entering the arenaceous wall, followed by a regular curve and a local maximum. In agglutinated foraminifera, this specific grain arrangement has been suggested to optimize the secretion of cement, making the test robust and more resistant to probable external stresses [12, 15, 18, 28, 30, 32]. It is possible that the same occurs for *S*. *alveolata*, whose tubes need to persist in high-energy habitats and reduce the stress transmitted to worms inside the tubes [29]. Indeed, the composition of the proteic cement secreted by the polychaete to glue sand grains has been shown to be optimised for that purpose [52, 53]. Nevertheless, differences in the fractal models were also observed. In *S*. *alveolata*, a few maxima are superimposed on a broad negative parabolic trend, whereas in *K*. *novangliae* ripples are more evident (Fig 5). The convergence of the building strategy of the metazoan tube and the protozoan test in terms of high selectivity in agglutinating grains and complexity in the agglutinated wall microstructure could result from the need of both taxa to optimise cost-benefits [56]. However, the analysis of the Hausdorff’s fractal dimension highlights differences in the agglutination strategies. Although the range of dimensions is similar, featuring values between 1.4 and 1.8 in both cases, and some similarities are observed in the trends of dimension vs. radial distance from the tube axis or chamber inside, the current evidence is still quite weak to state a true convergence based on fractal dimension alone. Many more analyses of arenaceous tubes of other polychaete species and agglutinated foraminifera (also belonging to different genera) should be carried out, possibly also considering different definitions of fractal dimension and different sizes of the computation window. Intraspecific differences within and among populations would also be interesting to explore, in order to recognise possible adaptations to local environmental contexts.

In the past, fossil agglutinated foraminiferal tubes, such as the giant genus *Bathysiphon* Sars, have been confused with those of polychaetes [e.g., 57]. The analysis of the Hausdorff’s fractal dimension performed on SEM images of sectioned agglutinated walls could be a useful tool to help palaeontologists differentiate worm tubes from tubular tests built by agglutinated foraminifera.

### First record of “plasticagglutinated” reefs from the Sicily channel

Plastic waste and microplastic pollution are ramping up, to the point that they can now be considered a “planetary boundary threat” capable of destabilizing the Earth’s normal function [58]. Every year, millions of metric tons of plastic debris from land reach the sea [59], where they may accumulate in marine sediments, thus entering in Earth’s sedimentary record [60], or may float in the water column and be ingested by marine organisms, thus clogging their digestive tract and entering the food chain [61–63]. Several works have documented the presence of micro- and nanoplastics incorporated into the soft tissues of marine invertebrates and vertebrates, such as seaworms, [e.g. 64], bivalves [e.g. 65], anthozoan corals [66, 67], marine sponges [68], sand fishes [69], and also unicellular organisms, such as benthic foraminifera [70–72]. So far, microplastic particles have been rarely observed in mineralized cases of aquatic organisms, as in caddisflies [freshwater insects of the order Trichoptera; 73, 74] and polychaete worms [e.g. 75], although these agglutinated cases could reasonably act as traps for microplastic debris, mostly in the coastal marine environment, where microplastic fragments are concentrated [e.g. 76–78].

Microplastics in seaworm tubes were observed for the first time in 2018, in the polychaete *Gumarea gaimardi* (Quatrefages, 1848) from South Africa [75], and more recently in *Sabella spallanzanii* (Gmelin, 1791) from the Italian coast on Western Mediterranean Sea [78], *Galathowenia* spp. and *Owenia borealis* Koh, Bhaud & Jirkov, 2003 from Norway and the Barent Sea [79] and *Phragmatopoma caudata* Krøyer in Mörch, 1863 from Brazil [80]. Our work reports for the first time the presence of microplastics agglutinated in the arenaceous bioconstructions of the honeycomb seaworm *S*. *alveolata*, from the Sicily channel (Central Mediterranean Sea). In this area, there are not big coastal cities or rivers that discharge large amounts of plastic debris. The top source of plastic pollution here is the litter discarded along shipping lanes, although it is also likely that floating plastics tend to be transported elsewhere by the Atlantic-Ionian Stream, a strong free-jet current mainly flowing eastward along the southern coast of Sicily [81]. Yet, the two study sites in Southern Sicily are located near a large marina and close to beaches highly attended by tourists, therefore local concentration of microplastics in the sea could be considerable.

It is probable that *S*. *alveolata* picked up microplastic fragments from seawater together with all other suspended particles and then agglutinated them in the tube, but we do not know whether their incorporation within the tube walls was accidental or intentionally operated by the polychaete worm, and whether this will produce adverse effects in the future [79, 80]. Microplastics are known vectors for microorganisms, pathogens and viruses [82, 83]. They could accumulate or react with harmful substances from the surrounding water, thus leaching toxic compounds into the environment and causing harm to marine animals [84–86]. While building the bioconstruction, sabellariid worms produce chemical signals that are responsible for larval settlement [87], but it is unknown whether microplastics could interfere with this process, causing a progressive loss of new individuals, thus hampering the building of the bioconstruction. Furthermore, if abundantly accumulated inside the arenaceous tubes, microplastic debris could alter the mechanical resistance of the reef to wave action, favouring bioconstruction erosion mostly during the winter season. Reef building polychaetes are ecosystem engineers that play a critical function in shallow water ecosystems, contributing to create and maintain habitats for several organisms and to control coastal erosion, stabilizing sandy sediments [e.g. 88–90]. The increasing abundance of this pollutant could represent a yet underestimated threat for such a fragile microhabitat. This calls for further, in-depth investigations, especially in coastal regions with high concentration of marine microplastics.

## Conclusions

In this work we were able to demonstrate that the honeycomb worm *Sabellaria alveolata* selects grains based on size and shape to build its arenaceous tubes, leading to different tube layers displaying distinct mineralogical compositions. With respect to previous works that performed similar analyses on the agglutinated surface of tubes [e.g. 29], or utilized thin sections observed at the polarized light microscope [e.g. 44, 91], we used polished sections of aggregated tubes analysed through a SEM equipped with EDS as in mineralogical and petrographic studies. This approach presents a number of advantages: (i) it documents the structure of aggregated tubes (e.g. the internal morphologies and the space between the different tubes); (ii) it evaluates wall thickness and grain distribution within the agglutinated wall, studying the grains in their original position within the wall and during tube building; (iii) it detects the chemical composition of the agglutinated grains in order to highlight compositional grain selectivity and preferential arrangement; (iv) it defines the elemental compositional data, and (v) it provides high resolution images that can be used to analyse the spatial distribution of agglutinated grains through mathematical modelling.

Moreover, this work presents a mathematical analysis (e.g. the Hausdorff fractal dimension) to compare the construction patterns used to agglutinate grains and form an external arenaceous case in two phylogenetically distinct organisms (a metazoan and a protozoan). In both organisms, the distribution of the agglutinated grains according to a layered fabric matches a fractal model that is probably adopted to optimize the production of cement and strengthen the arenaceous wall.

This study also documents for the first time the presence of microplastics agglutinated in the tubes of *Sabellaria alveolata*. This unexpected finding calls for a broad-scale study of microplastic particle inclusion within shallow coastal bioconstructions, in order to assess how widespread a phenomenon this is.

## Supporting information

S1 Fig Scheme with the bioconstruction sections prepared and analysed for the present work. Five horizontal and 4 vertical sections of bioconstruction from the two studied sites (Santa Barbara–SB and Porto Turistico–PT) were prepared as polished sections and analysed through a SEM equipped with EDS.
(TIF)

S2 Fig Back-scattered electron (BSE) image of aggregated tubes (the same of Fig 1; photo 1) that has been horizontally sectioned in the middle (section 5B) and at the top (section 5A), respectively. The coloured crosses indicate the spots for standardless microanalyses; the corresponding EDS spectra as well as the elemental maps are reported below.
(ZIP)

S3 Fig BSE image of aggregated tubes (the same of Fig 1; photo 2) that has been vertically sectioned (section 6A). The coloured crosses indicate the spots for standardless microanalyses; the corresponding EDS spectra as well as the elemental maps are reported below.
(TIF)

S4 Fig 1) SEM image in secondary electrons of Sabellaria alveolata bioconstruction from site SB (horizontal view showing the typical honeycomb structure). 2–5) BSE images of aggregated tubes (the same of photo 1) that have been vertically sectioned (sections 1A and 1B). The elemental maps are reported below. Noteworthy is the disposition and composition of the agglutinated grains that does not changed in the bioconstructions from the two studied sites.
(ZIP)

S5 Fig BSE images of aggregated tubes from Santa Barbara site that has been vertically sectioned (sections 2A and 2B). The elemental maps are reported below.
(ZIP)
