# Supplementary figures and images for: Fractal analysis highlights analogies in arenaceous tubes of Sabellaria alveolata (Metazoa, Polychaeta) and agglutinated tests of foraminifera (Protista)
Source: PLoS One. 2022 Aug 26;17(8):e0273096. doi: 10.1371/journal.pone.0273096 (PMC9417037; doi:10.1371/journal.pone.0273096)

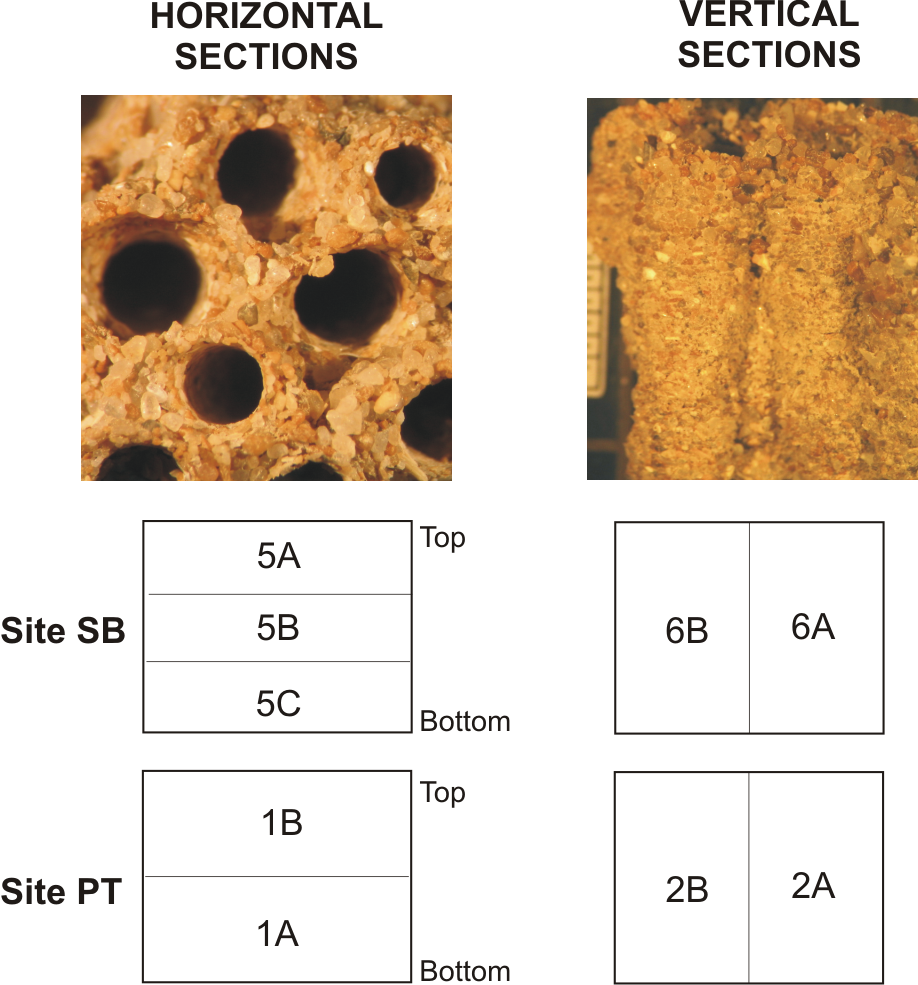

Supplement: S1 Fig — Five horizontal and 4 vertical sections of bioconstruction from the two studied sites (Santa Barbara–SB and Porto Turistico–PT) were prepared as polished sections and analysed through a SEM equipped with EDS. (TIF) [file pone.0273096.s001.TIF]

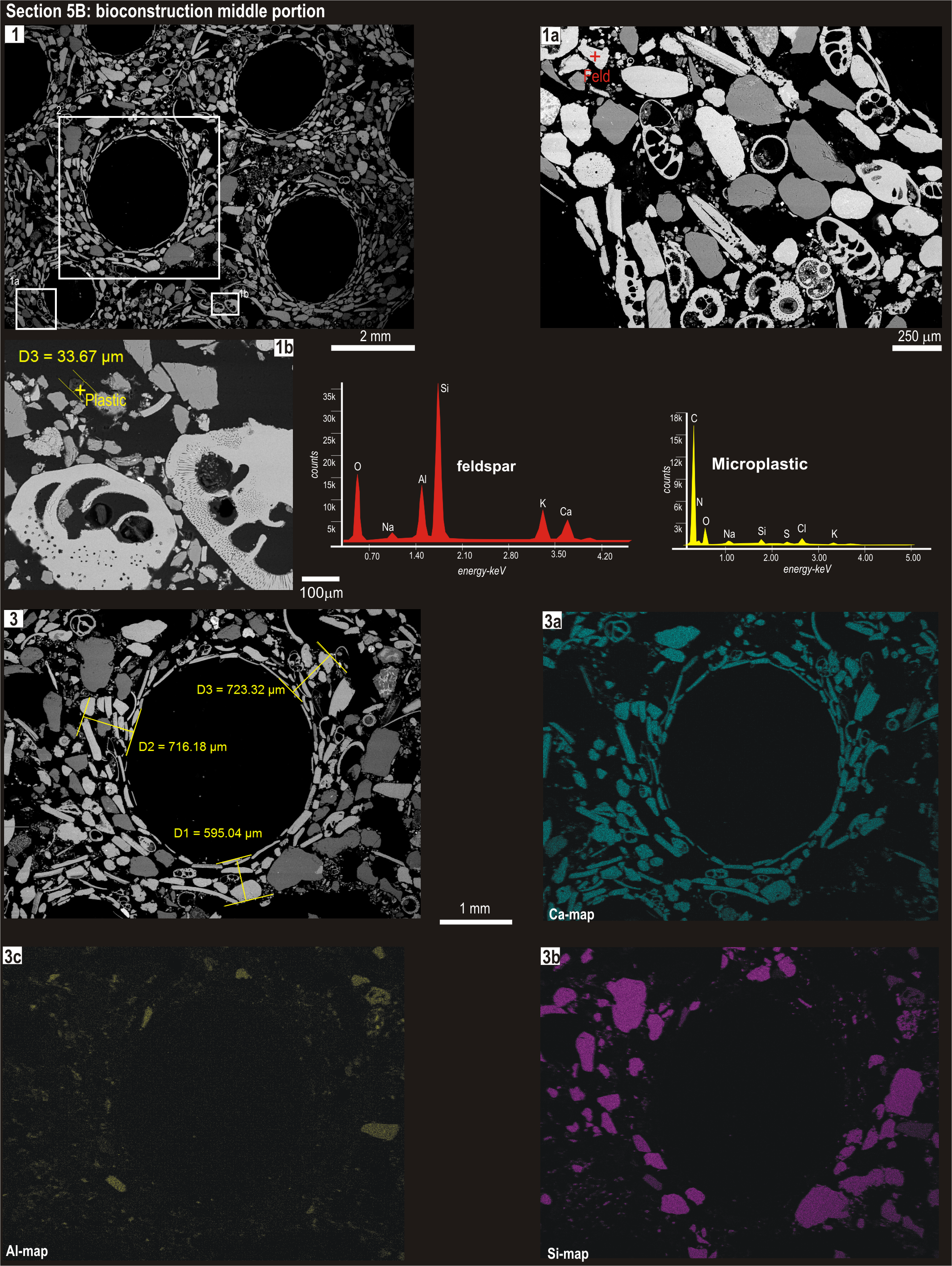

Supplement: S2 Fig — The coloured crosses indicate the spots for standardless microanalyses; the corresponding EDS spectra as well as the elemental maps are reported below. (ZIP) [file pone.0273096.s002.zip › S2 Fig.tif]

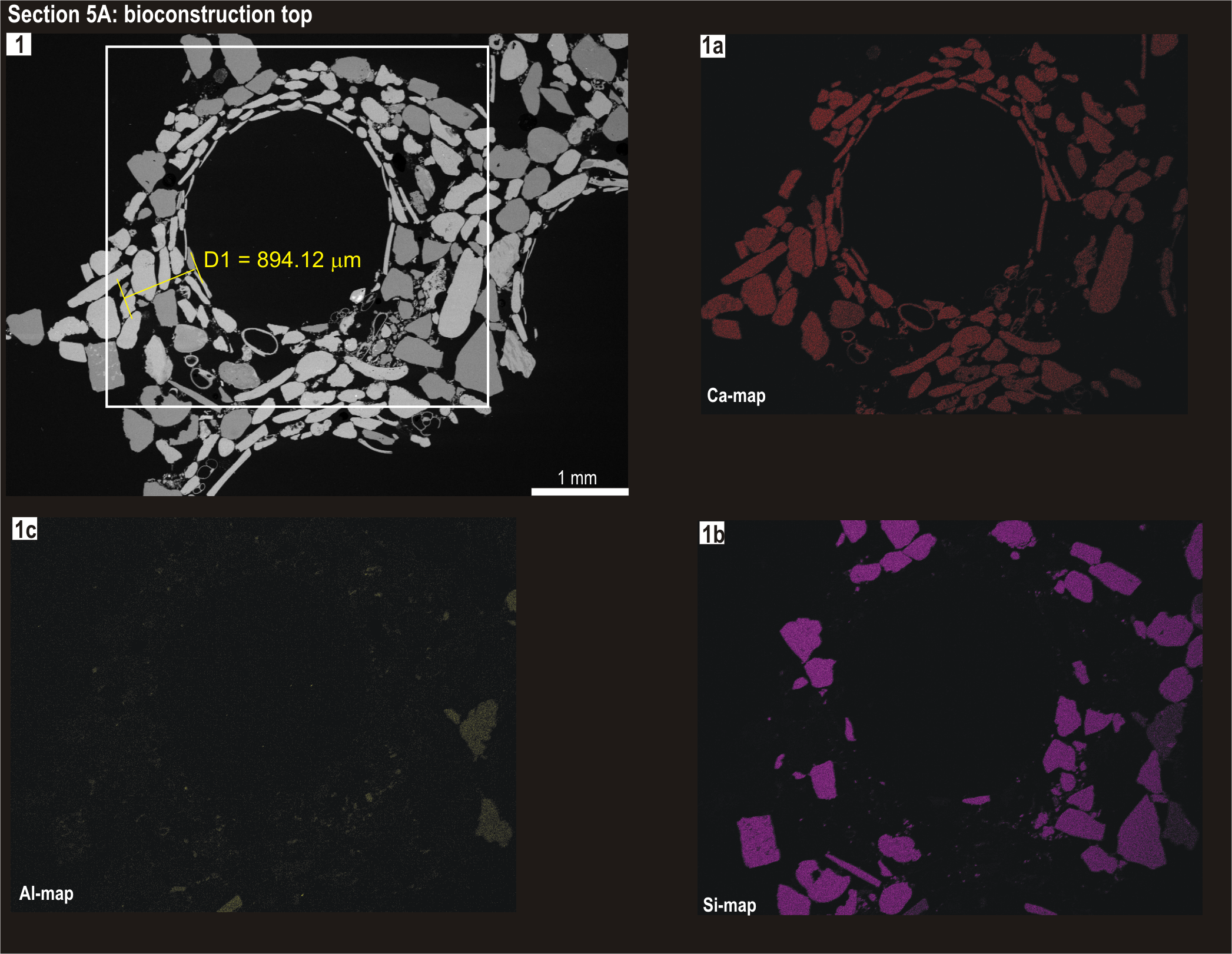

Supplement: S2 Fig — The coloured crosses indicate the spots for standardless microanalyses; the corresponding EDS spectra as well as the elemental maps are reported below. (ZIP) [file pone.0273096.s002.zip › S2 Fig-continued.tif]

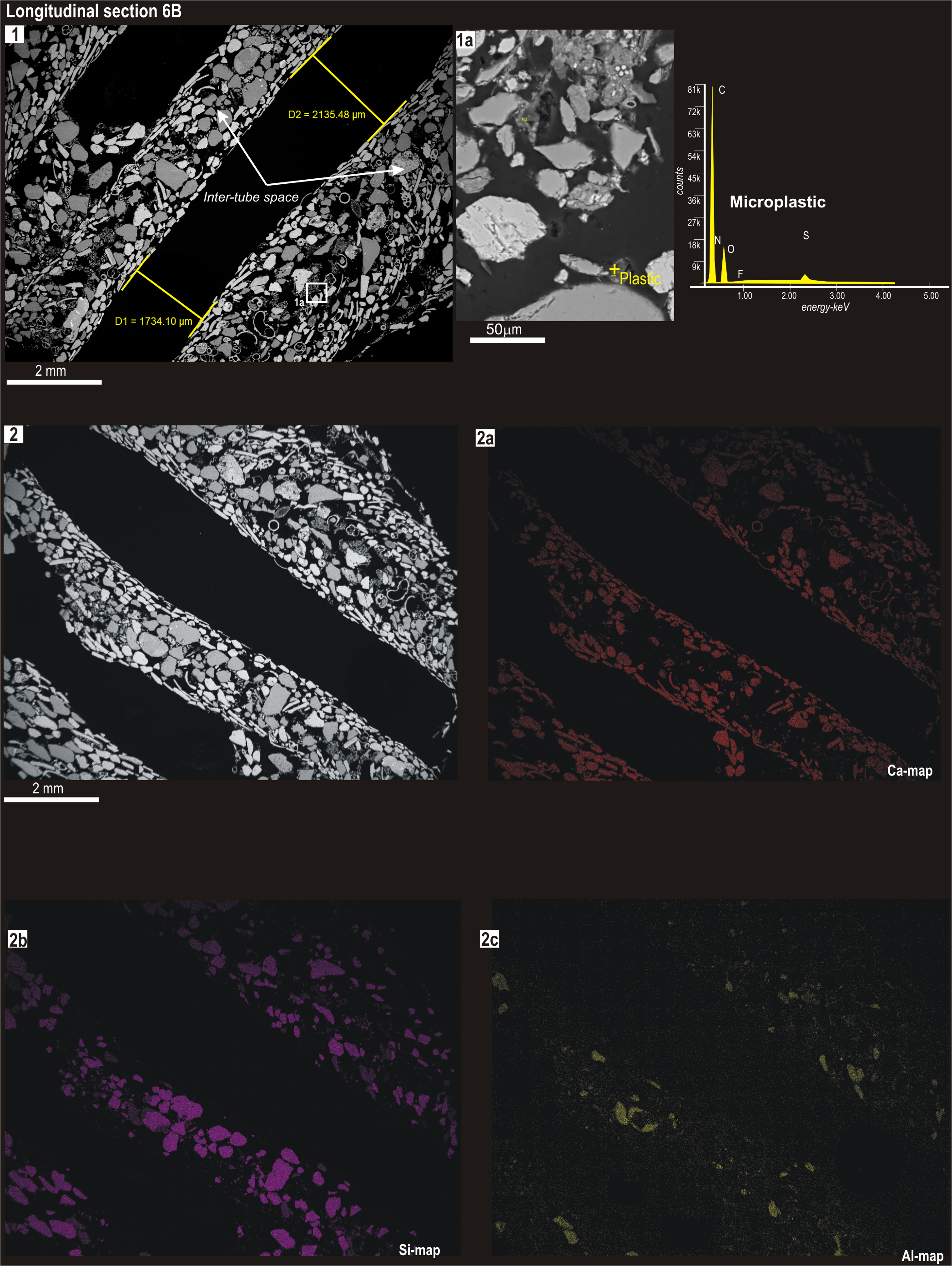

Supplement: S3 Fig — The coloured crosses indicate the spots for standardless microanalyses; the corresponding EDS spectra as well as the elemental maps are reported below. (TIF) [file pone.0273096.s003.tif]

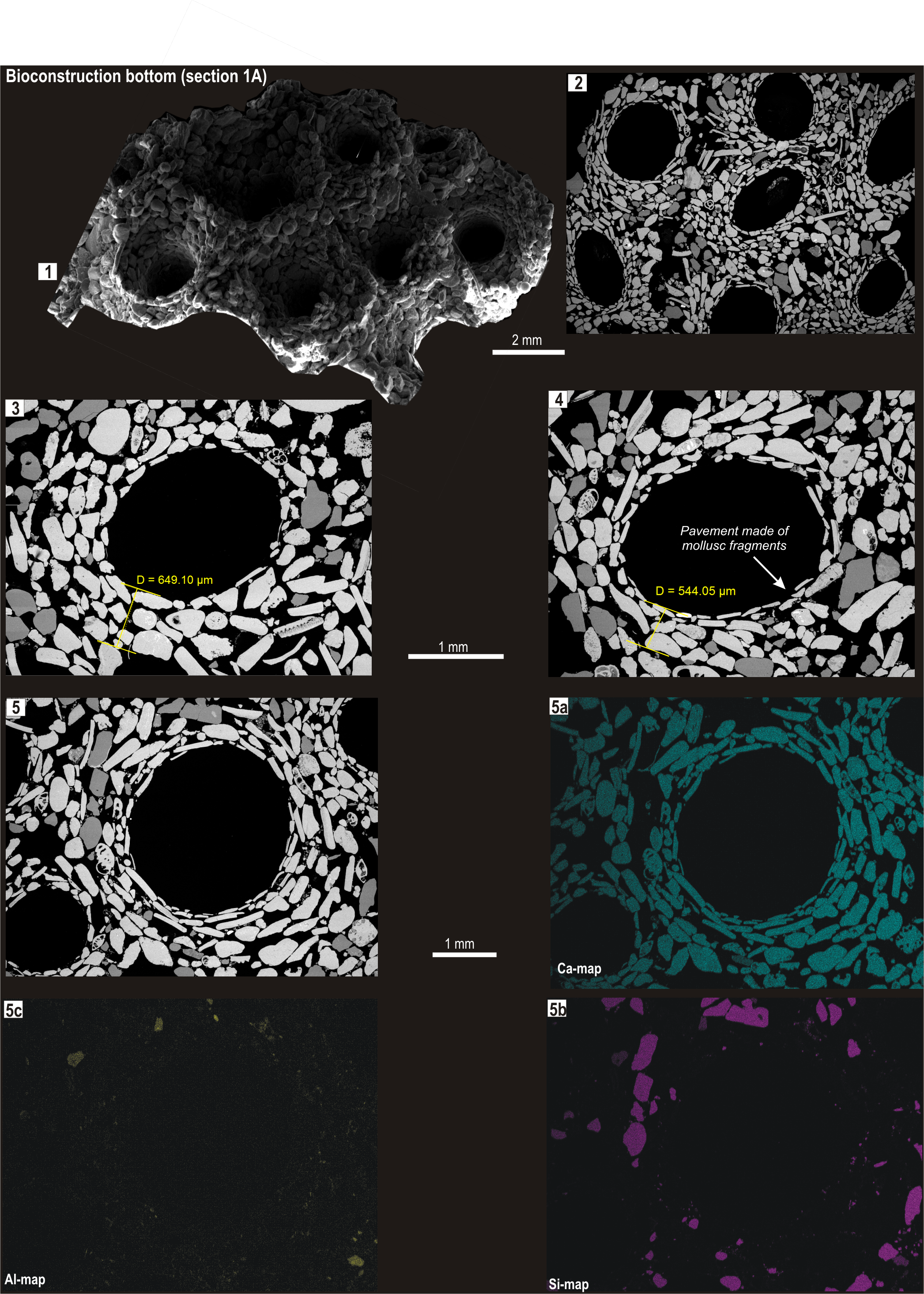

Supplement: S4 Fig — 2–5) BSE images of aggregated tubes (the same of photo 1) that have been vertically sectioned (sections 1A and 1B). The elemental maps are reported below. Noteworthy is the disposition and composition of the agglutinated grains that does not changed in the bioconstructions from the two studied sites. (ZIP) [file pone.0273096.s004.zip › S4 Fig.tif]

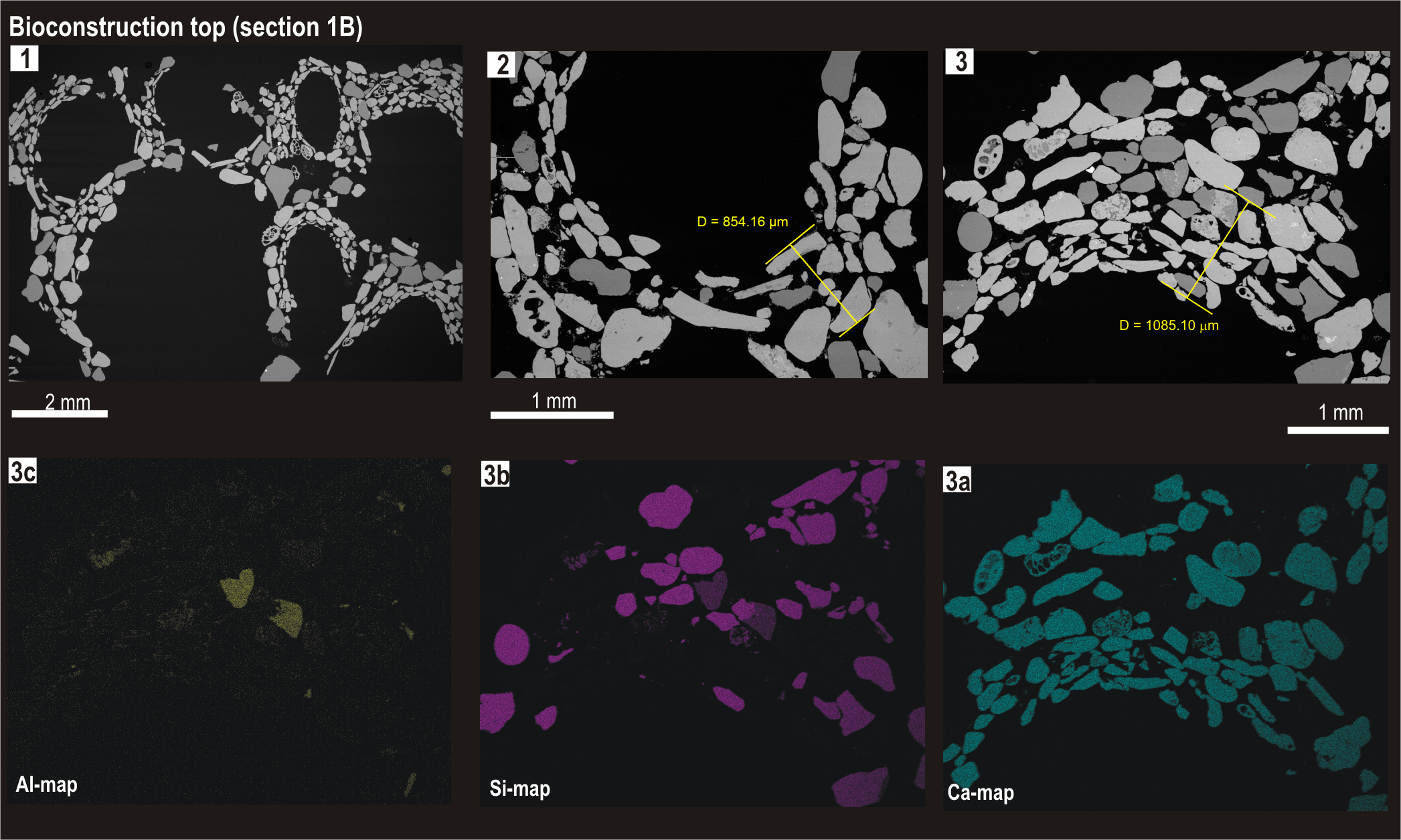

Supplement: S4 Fig — 2–5) BSE images of aggregated tubes (the same of photo 1) that have been vertically sectioned (sections 1A and 1B). The elemental maps are reported below. Noteworthy is the disposition and composition of the agglutinated grains that does not changed in the bioconstructions from the two studied sites. (ZIP) [file pone.0273096.s004.zip › S4 Fig-continued.tif]

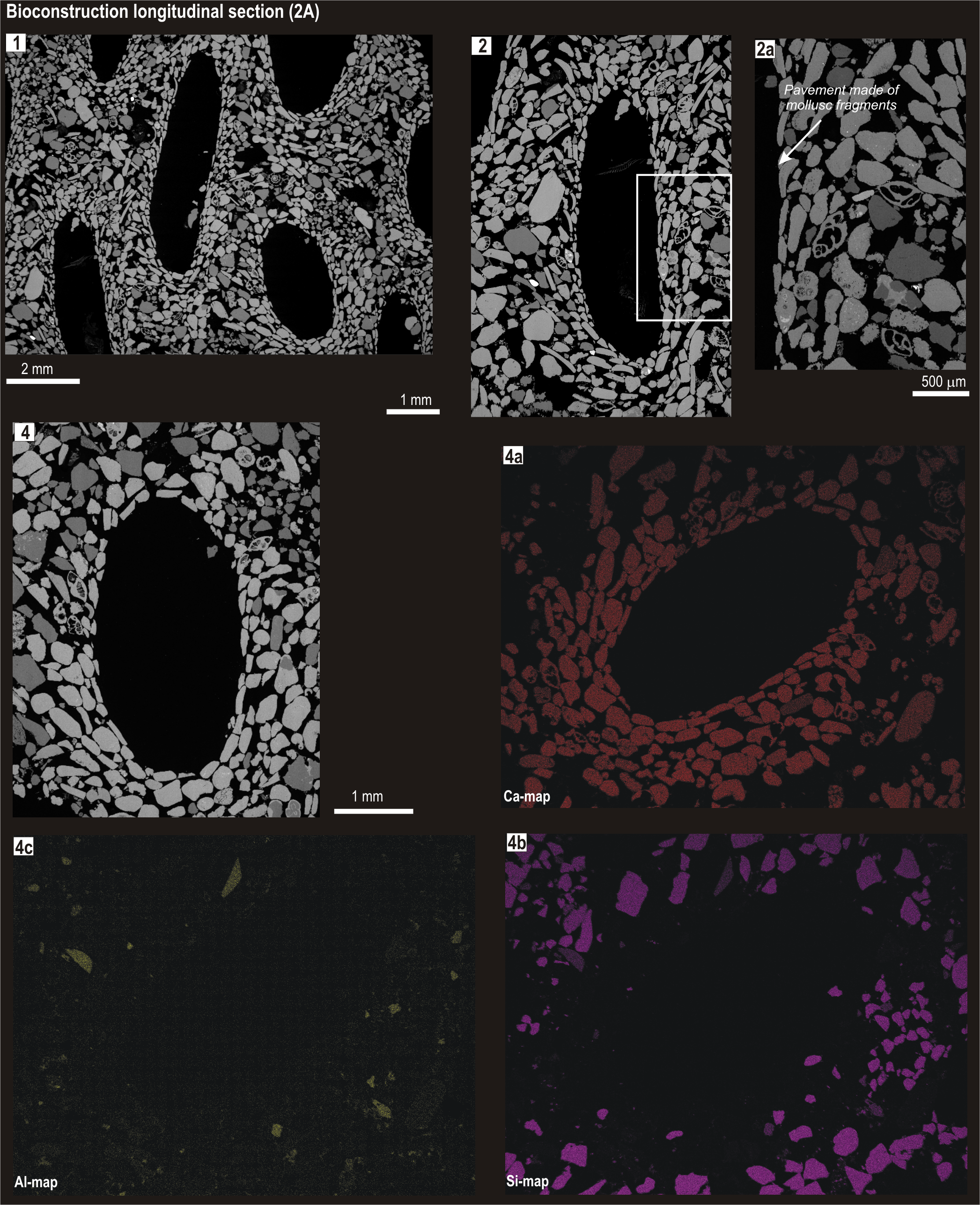

Supplement: S5 Fig — The elemental maps are reported below. (ZIP) [file pone.0273096.s005.zip › S5 Fig.tif]

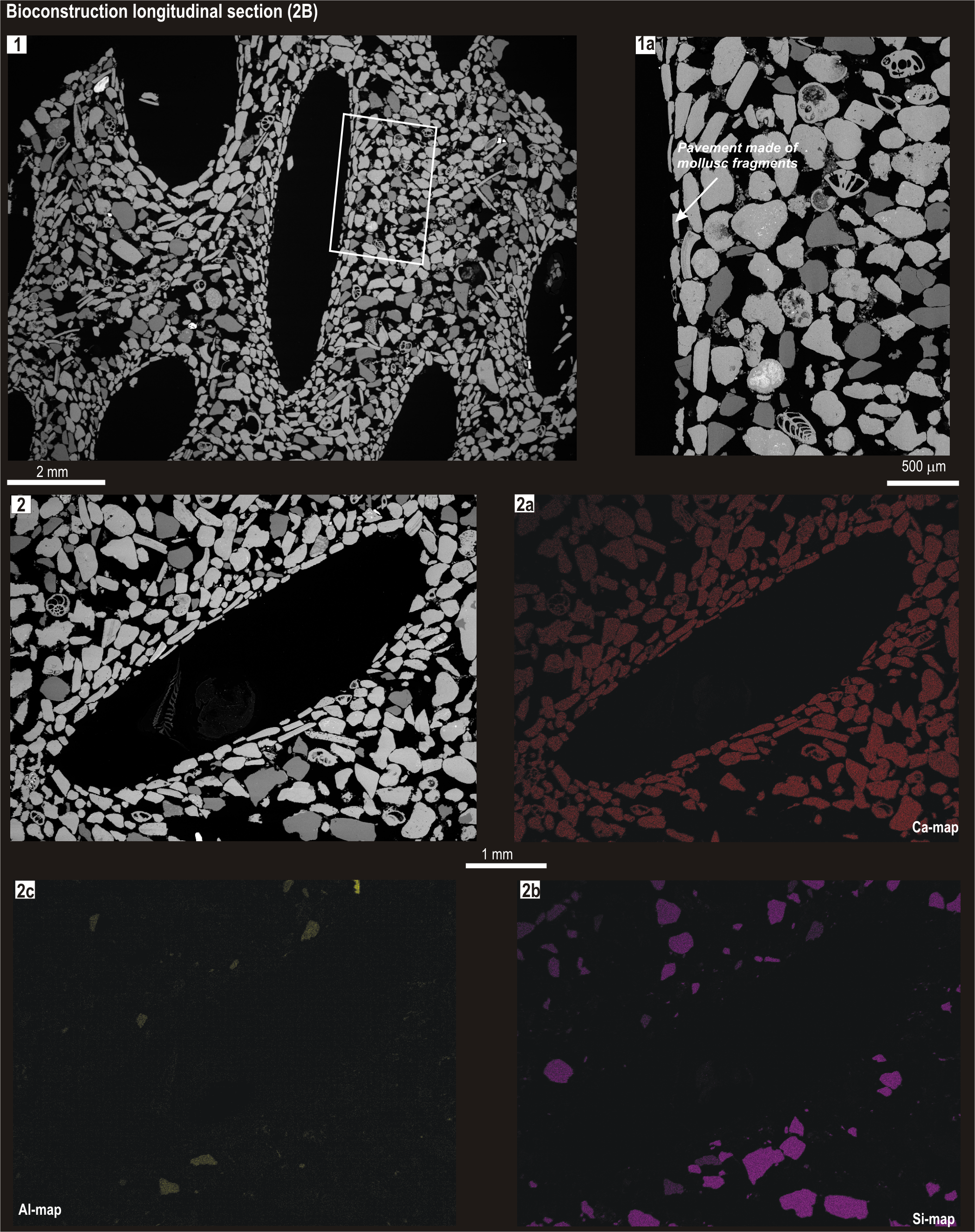

Supplement: S5 Fig — The elemental maps are reported below. (ZIP) [file pone.0273096.s005.zip › S5 Fig-continued.tif]
